# Diversity of antimicrobial resistance and virulence genes of pathogenic *Escherichia coli* recovered from pigs in England

**DOI:** 10.3389/fmicb.2025.1668327

**Published:** 2025-10-22

**Authors:** Samuel A. M. Connelly, Manal AbuOun, Nicholas Duggett, Miranda Kirchner, Indre Navickaite, Javier Nunez-Garcia, Susanna Williamson, Kelly Vaughan, Christopher Teale, Muna F. Anjum

**Affiliations:** ^1^Animal and Plant Health Agency, Weybridge, United Kingdom; ^2^Animal and Plant Health Agency, Thirsk Veterinary Investigation Centre, Thirsk, United Kingdom; ^3^Animal and Plant Health Agency, Bury St Edmunds, United Kingdom; ^4^APHA Veterinary Investigation Centre, Exeter, United Kingdom; ^5^Veterinary Investigation Centre, Animal and Plant Health Agency, Shrewsbury, United Kingdom

**Keywords:** *Escherichia coli*, antimicrobial resistance, virulence, postweaning diarrhoea, pig health, whole genome sequencing

## Abstract

**Introduction:**

We characterised the genomes of 208 pathogenic *Escherichia coli* isolated from pigs diagnosed with enteric colibacillosis (EC), bowel oedema (BO) or colisepticaemia (CS) in England between 2016–2020 to investigate their antimicrobial resistance (AMR) and virulence-associated genes (VAGs).

**Methods:**

AMR and VAGs were identified using the APHA SeqFinder tool, while SNP comparisons were performed with Snippy and RAxML-NG. A subset of isolates were long-read sequencing to examine mobile genetic elements.

**Results:**

The EC group primarily carried fimbrial and toxin genes, including *faeG* (F4, 33%) and *stb* (STb, 48%). The BO group primarily encoded *fedF* (F18, 38%) and *stx2e-AB* (Stx2e, 38%). The CS group were more homogeneous, with over 70% harbouring genes for curli fibre (*csgABGFE*), haemolysin, *iss*, iron transport (*sitABCD*), and siderophore (*iucABCD*, *iroBCDEN*) genes. Overall, 62% of isolates were multidrug resistant (MDR) with the Enterotoxigenic *E. coli* (ETEC) showing an MDR prevalence of 73%. Phylogenetic analysis identified two high-risk sequence types (STs), ST90 and ST772; both unique to juvenile pigs and previously under-reported in the UK. Plasmid analysis of representative ETECs from these STs revealed large MDR plasmids potentially co-linked with metal and disinfectant resistance genes.

**Discussion:**

This study highlights the predominant AMR and VAGs in pathogenic *E. coli* from diseased pigs in England, the emergence of high-risk lineages, and the role of mobile genetic elements in resistance dissemination. These findings improve understanding of pathogenic *E. coli* in pigs and support efforts to improve diagnostics, guide treatment, and control of farm outbreaks.

## 1 Introduction

*Escherichia coli* are Gram-negative, rod-shaped bacteria that make up part of the commensal microbial population of the vertebrate gut (Tenaillon et al., 2010). Some types of *E.coli* are opportunistic pathogens causing various pathologies in their hosts, ranging from diarrhoeal disease to sepsis, depending on the carriage of particular virulence associated genes (VAGs) (Tenaillon et al., 2010).

Pathogenic *E. coli* can be broadly split into intestinal pathogenic *E. coli* (InPEC) and extraintestinal pathogenic *E. coli* (ExPEC), based on location of the host tissue where they induce disease. Both groups can be characterised into the 11 pathotypes described, based on their phenotypic characteristics (Geurtsen et al., 2022).

Within the food chain, pathogenic *E. coli* causes a particular problem for juvenile pigs. Young pigs (up to 20 weeks) have immature immune systems, and early in life they rely on the passive immunity provided by the sow’s colostrum to protect them from bacterial infections, including pathogenic *E. coli* (Fairbrother and Nadeau, 2019). But due to naivety of the immune systems of juvenile pigs, there is an increased risk of morbidity and mortality associated with the pre- and post-weaning periods. Resulting outbreaks incur financial costs to farmers, decrease animal welfare, and increase challenges to the UK pork industry (Fairbrother and Nadeau, 2019).

InPEC frequently cause enteric colibacillosis (EC) infections in UK herds (APHA and SRUC, 2024), which is typically caused by the enterotoxigenic *E. coli* (ETEC) pathotype. This pathotype is characterised by expression of one or more fimbriae or adhesins, which facilitate adherence to epithelial cells of the intestine. In Europe, the predominant fimbriae associated with ETEC are F4 (previously K88) and F18, with much lower abundance of the F5, F6 and F41 fimbriae (Luppi et al., 2016). ETEC also secrete one or more enterotoxins which include the heat-labile (LT) and the heat-stable (STa and STb) toxins (Fairbrother and Nadeau, 2019; Fairbrother et al., 2005). The combination of these VAGs causes the release of ions and water into the gut lumen, resulting in bouts of pre- and post-weaning diarrhoea (PWD) (Duan et al., 2019; Turner et al., 2006), leading to increased morbidity and mortality.

InPEC are also responsible for bowel oedema (BO) due to vascular damage, where oedematous change may be visible in the submucosa of the gastrointestinal tract, subcutis, and brain. This is caused by the Shiga-toxin producing *E. coli* (STEC), which carry a prophage harbouring genes for the Stx1 and/or Stx2 Shiga-toxins (Rodriguez-Rubio et al., 2021). Typically, porcine STEC carry the Stx2e toxin variant whilst simultaneously expressing the F18 fimbriae on their surface (Fairbrother and Nadeau, 2019). Outbreaks of BO may also occur concurrently with or independently of, outbreaks of EC (Fairbrother and Nadeau, 2019).

There have also been reports of “hybrid” InPECs, incorporating genetic markers from both STEC and ETEC in humans (Lee et al., 2023) and animals (Zhang et al., 2007). The carriage of these genetic markers on mobile genetic elements (MGEs) gives them the potential to move both horizontally and vertically through bacterial populations. These hybrid pathotypes can have variable phenotypes which can make pathotyping more challenging (Robins-Browne et al., 2016).

The ExPEC group are a less well-defined group of disease-causing *E. coli* and are opportunistically invasive (Robins-Browne et al., 2016). These *E. coli* are usually indistinguishable from commensal *E. coli* of the gut, and do not have a well-defined group of virulence factors. Instead, they can harbour many different virulence factors that contribute to disease (Fairbrother and Nadeau, 2019; Geurtsen et al., 2022). In this study, we focus on ExPECs associated with colisepticaemia as it primarily causes disease in neonatal or pre-weaned piglets. CS is often caused by ExPEC that encode virulence factors supporting their survival in the bloodstream, such as the bacteriocin, colicin V and aerobactin (Mokady et al., 2005; Pitout, 2012).

Options for treatment using authorised antimicrobials can be limited or unsuccessful due to antimicrobial resistances that may be present in *E. coli* from pigs (AbuOun et al., 2020). There is also a requirement to avoid medically or veterinary important antimicrobials in livestock, so future treatments are not compromised (Luppi, 2017; Gale and Velazquez, 2020; World Organisation for Animal Health, 2021; World Health Organisation, 2024). However, antimicrobial sensitivity of the *E. coli* pathogen may not be known before treatment is initiated, and any antimicrobial use risks selection of AMR bearing organisms.

Previous studies have reported on AMR in commensal *E. coli* from healthy pigs (AbuOun et al., 2020), but there is limited information on AMR in pathogenic *E. coli* from diseased pre- and post-weaned pigs, where antimicrobial usage may be higher in response to infection control measures in herds. To address this, we used whole genome sequencing (WGS) to characterise the AMR and VAGs present in 208 *E. coli* isolated from pigs in England and Wales from between 2016 and 2020, with a confirmed diagnosis for one of the three pig-associated *E. coli* diseases (EC, BO or CS) described above. We then carried out phylogenetic analysis to compare isolates from this study to understand their genetic relatedness. We then contextualised these isolates with published data from our group for *E. coli* isolated from healthy pigs and pigs at slaughter. This highlighted some differences between *E. coli* isolated from difference stages of the food production system. Finally, long-read sequencing was used to elucidate the MGEs from a subset of isolates, allowing us to identify AMR bearing plasmids, and associate these with certain lineages. Together, these data will help improve our understanding of pathogenic *E. coli* associated with infections in pigs and their treatment.

## 2 Materials and methods

### 2.1 Selection, culturing, and DNA extraction from bacterial isolates

Two hundred and eight samples submitted between 2016 and 2020 primarily from England (*n* = 203/208) and a small number from Wales (*n* = 5/208), were selected from the APHA pig scanning surveillance archive. Isolates were cultured from samples originating from neonatal (<1 week), pre-weaned (6 days - 8 weeks) and post-weaned (>1 month) pigs that had been diagnosed with either EC, BO, or CS. A full list of isolates can be found in Supplementary Table 1.

For the enteric *E. coli*, a sterile swab was used to culture bacteria from either the intestines or faeces. For the invasive CS isolates, a tissue sample such as liver, spleen, meninges, and others (excluding intestinal or faecal samples) was seared to sterilise the surface, and a swab was taken from the incision site. Swabs were spread onto MacConkey agar after an overnight incubation at 37 °C, and then a single lactose-fermenting (pink) colony subcultured to MacConkey and grown under the same conditions. Isolates were presumptively identified as *E. coli* if oxidase negative, catalase positive and indole positive.

Isolates submitted between 2016 and 2017 from animals presenting symptoms of either EC or BO were also subjected to an in-house real-time PCR (qPCR) to validate a real-time PCR that detects nine virulence genes associated with ETEC and STEC (*faeG*, *fedA*, *fim41a*, *fanA*, *fasA*, *f17A*, *eltA*, *sta1* and *stx2e*). This PCR was implemented in the routine diagnostics of ETEC and STEC at the APHA in 2021 which is why further isolates from this study were not included in the panel. The oligos and reaction conditions for this real-time PCR can be found with document in the Supplementary materials. Comparative analysis of the results from the real-time PCR can be found in Supplementary Table 1. The data from this qPCR was also validated against the WGS data generated in this paper.

Purified isolates were stored on beads at –80 °C until needed. A 1 μL loop of frozen stock was subcultured onto Luria Bertani (LB) agar and grown overnight at 37 °C. A single colony was selected and inoculated into LB broth and grown overnight at 37 °C, whilst shaking at 200 revolutions per minute, for DNA extraction.

DNA was extracted from overnight cultures using the Applied Biosystems™ MagMax™ CORE Nucleic Acid Purification Kit (Thermo Fisher Scientific, United Kingdom) with the Thermo Fisher Scientific KingFisher Flex System as per the manufacturer’s instructions (Thermo Fisher Scientific, United Kingdom). Isolates for long read sequencing were cultured overnight in the same way as above and extracted using the PDQeX nucleic acid extractor (MicroGEM, United Kingdom) as per the manufacturer’s instructions.

### 2.2 Sequencing and statistical analysis

Short-read sequencing was carried out using the Illumina NextSeq system as previously described (AbuOun et al., 2021). The data for this project is stored in the European Nucleotide Archive (Accession: PRJEB77416). For long-read sequencing, four isolates were sequenced on the Oxford Nanopore Technologies (ONT, United Kingdom) MinION system (Accession: PRJEB97106) using the Rapid barcoding kit (SQK-RBK004; ONT, United Kingdom). The sequencing library was prepared following manufacturer’s instructions with the exception that the AMPure XP bead (Beckman Coulter, United States) incubation was carried out on a Hula mixer™ (ThermoFisher Scientific) for 20 min. Samples were eluted in 10 mM Tris–HCl pH 7.5–8.0 with 50 mM NaCl whilst incubated at room temperature for 5 min. Sequencing was then carried out using R9.4 flow cells and base calling performed using Guppy (v5.0.17) with default parameter settings and the quality threshold set to 9. Long-read sequencing for isolate 149–258 was repeated using the Rapid barcoding kit 24 V14 (SQK-RBK114.24; ONT, United Kingdom) with the R10.4.1 flow cells, and Guppy (v6.5.7) to improve genomic resolution for this isolate.

Genomes were assembled using the Unicycler v0.4.8 pipeline (Wick et al., 2017), and hybrid assemblies created where short and long-read data was available. The quality of the raw and assembled data was checked using the FastQC v0.11.8,^1^ multiQC v1.10.1 (Ewels et al., 2016), and QUAST v5.1 tools (Gurevich et al., 2013). Kraken2 v1.1.1 (Wood et al., 2019) was used to assign taxonomic groupings to the raw reads and isolates that failed any of the QC checks were re-sequenced.

The APHA SeqFinder software (Duggett et al., 2020) was used to map sequence reads to reference database for AMR (AbuOun et al., 2021) and VAGs (Anjum et al., 2007; Wu et al., 2008). We also used a custom database to identify heavy metal and biocide resistance genes that was adapted from the BacMet database (Pal et al., 2014). ABRicate v0.8^2^ was used to verify the locations of genes within contigs of assembled data.

VAGs included in the main body of text based on their prominence in the literature. When assigning pathotypes, we used a previously published scheme for assigning ETECs and STECs (Luppi, 2017). For the ExPEC, we used the most common genes amongst this group, due to the homogeneity of their VAGs.

AMR genes that confer resistance to antimicrobial classes that were not listed in the World Organisation for Animal Health (WOAH) list of antimicrobial agents of veterinary importance were excluded from this study (World Organisation for Animal Health, 2021), including genes for hygromycin and streptothricin. We did include the *catA* and *cml* genes for chloramphenicol resistance in this list due to chloramphenicol’s classification as a highly important antimicrobial (HIA) in humans (World Health Organisation, 2018). Multidrug resistance (MDR) was determined based on the presence of resistance genes to three or more classes of antimicrobials.

Statistical analyses were conducted in Microsoft Excel 365, where Pearson’s chi-squared tests were used to identify significance between observed and expected values. R v4.0.4 was used for data visualisation with all plots generated using the ggplot2 package (Villanueva and Chen, 2019).

### 2.3 Phylogenetic comparisons between *Escherichia coli* from this study

The Roary tool was used to estimate the number of genes in the core and accessory genomes of the 208 *E. coli* from this study (Page et al., 2015). Multi-locus sequence types (MLST) were assigned using SRST2 v0.2.0 (Inouye et al., 2014) as previously described (AbuOun et al., 2021). Any ambiguous sequence types (STs) were confirmed with the pubMLST database. Phylogroups were determined using ClermonTyper (Beghain et al., 2018).

The snippy V3.1 tool (Seemann, 2015) was used to call variants from WGS reads and a core genome single nucleotide polymorphism (SNP) alignment was generated using isolate *E. coli* MG1655 U00096.3 as a reference. Analysis of SNP differences was carried out in R v4.5.1 (R Core Team, 2025) using the Tidyverse packages (Wickham et al., 2019).

RAxML-NG V1.0.3 (Kozlov et al., 2019) was used to generate a maximum-likelihood (ML) tree file with 100 bootstraps using the GTR + FO + G4m model of nucleotide substitution. We visualised the phylogenetic tree using the ggplot2 v3.5.2 (Villanueva and Chen, 2019) and ggtree v3.16.3 (Yu et al., 2016) packages for R. Phylogenetic and evolutionary analysis between clades was carried out using the ape v5.8-1 package (Paradis and Schliep, 2019).

We used the K-mer based comparison tool, Sourmash v4.8.12 (Pierce et al., 2019), to create a dissimilarity matrix of hash scores. Any isolates that had a dissimilarity score of ≥80% were removed from the analysis due to their likelihood of being an unrelated species. The Pheatmap (Kolde, 2018) and Tidyverse packages were used to hierarchically cluster and visualise the dissimilarity matrix in R. Metadata such as ST, phylogroup, pig age, host diagnosis, MDR genotype, and study were used to identify subclusters within the dataset.

### 2.4 Selection of publicly available datasets and their relationship to *Escherichia coli* from this study

To contextualise the pathogenic *E. coli* within this study with *E. coli* isolated through various stages of pig production, we chose to utilise two publicly available datasets previously generated through studies carried out by the APHA. *E. coli* sequences came from two datasets: the ARDIG dataset (Accession: PRJNA750276), containing *E. coli* sequence data that were initially isolated from five different age groups of healthy pigs (Storey et al., 2022); and the second, MOLSIG (Accession: PRJEB26317) dataset, containing sequence data of *E. coli* isolated from adult pigs at slaughter (AbuOun et al., 2020). To reduce selection bias, we also ensured that sequences were only used for *E. coli* genomes that had been grown on non-selective plates, as was the case with the 208 *E. coli* in this study.

### 2.5 Analysis of plasmids

Plasmids were annotated using Bakta v1.11.0 (Schwengers et al., 2021), and visualised in BRIG (Alikhan et al., 2011). Linear comparisons of genomic loci were visualised using the Easyfig v2.2.2 software (Sullivan et al., 2011). Images were edited in Inkscape (v1.2).

## 3 Results

### 3.1 Host background for pathogenic *Escherichia coli*

A total of 208 *E. coli* isolated from diseased pigs submitted to the APHA between 2016 and 2020 through its scanning surveillance programme^3^ were genomically characterised in this study. All pigs had a confirmed postmortem and laboratory diagnosis of one of the three *E. coli* related diseases: EC (*n* = 122, 59%), BO (*n* = 34, 16%) or CS (*n* = 52, 25%). A list of isolates and related metadata can be found in Supplementary Table 1.

### 3.2 Relative proportion of VAGs

Current practice at the APHA is to screen suspected ETEC and STEC isolates associated with EC and BO for some of the known virulence factors associated with enteric disease in juvenile pigs. This high-throughput and cheap alternative to sequencing allows us to make an initial assessment of which isolates should be taken forward for further analysis by WGS. During this study, we validated a qPCR to determine the congruence of genotype and phenotype for virulence (Supplementary Table 1). Approximately 97% (*n* = 95/98) of the *E. coli* screened by both the qPCR and WGS showed good congruence, supporting our use of these methods for an initial screen of isolates collected through scanning surveillance.

**Figure 1:**
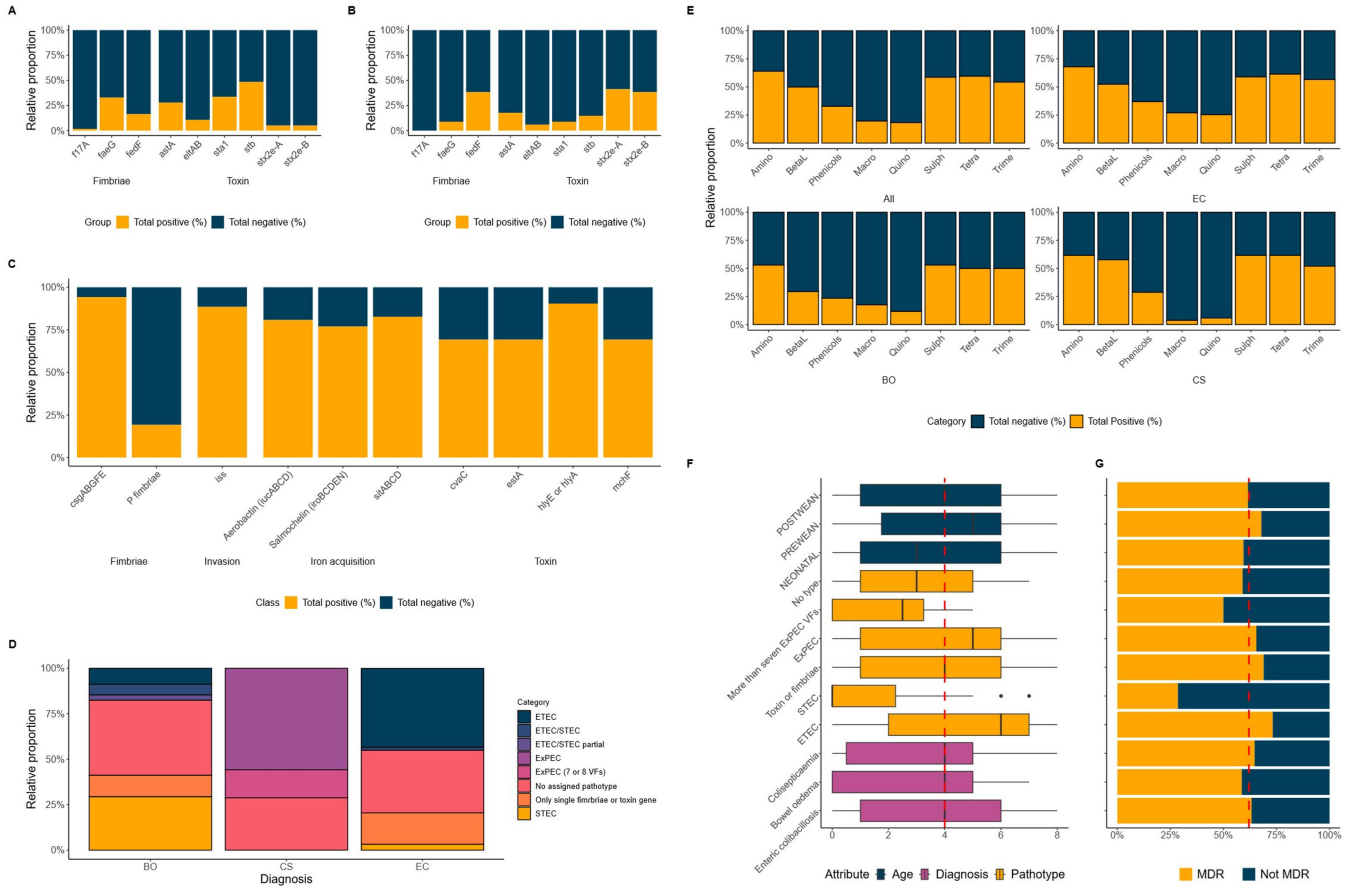
Panels **(A–C)** show the relative abundance of VAGs associated with ETEC, STEC and ExPEC for EC **(A)**, BO **(B)** and CS **(C)** groups of *E. coli*. Panel **(D)** shows the relative abundance of each pathotype the *E. coli* were grouped into based on their respective genotypes. Panel **(E)** shows the relative abundance of resistance genotypes for CIA and HIA classes for all, EC, BO and CS groups. The final panel **(F)** shows the median number of HIA and CIA classes for the enteric (EC and BO) and extraintestinal (CS) groups as well as pathotype alongside the prevalence of MDR **(G)**. The red dashed line represents the median or mean value.

Further work to broadly characterise the primary VAGs in these *E. coli* was then carried out. The VAGs associated with ETEC have been well defined in pigs (Luppi, 2017) and a full list of identified genes identified in these isolates is given in Supplementary Table 2. The genes, *faeG* and *fedF*, which encode components of the F4 and F18 fimbriae, were the most prevalent fimbrial genes identified in the EC (33%) and BO (38%) isolates, respectively (Supplementary Table 2). The *f17A* gene, which encodes the F17 fimbriae, was also present in a small population of EC (~2%) and CS isolates (~2%) (Figures 1A,B).

The genes encoding the LT (*eltAB*), STa (*sta1*), and the Stx2e (*stx2e-AB*) toxins were only found in the EC and BO associated *E. coli*. The *stb* gene (encoding STb) was the most prevalent toxin gene found in EC isolates (48%).

The most common toxin genes in BO isolates were the *stx2e-A* (41%) and *-B* (38%) subunit genes, with one isolate lacking the B-subunit gene. Additionally, a small population of the EC isolates also carried the *stx2e-AB* (5%) genes (Figures 1A,B). We observed that there was a statistically significant relationship between the presence of a complete *stx2e-AB* genes and disease state of the host (*p* value = 4.8 × 10^−6^). The chances of an isolate encoding the Stx2e toxin were higher in *E. coli* associated with BO than EC (OR = 7.8, CI = 2.9, 20.5). We also note, there was a statistically significant relationship between the presence of the F18 fimbriae genes, *fedA* and *fedF*, with the *stx2e-AB* genes (*p* value = 7.6 × 10^−10^).

We included a variety of VAG targets for the CS group of *E. coli* which included factors for adhesion, invasion, iron acquisition and toxin production. There was less heterogeneity of the VAGs within this group, with >70% of isolates carrying the most common genes, including the *csgABGFE* (curli fibre) genes present in virtually all CS isolates (94%), haemolysin (90%) and *iss* (88%) genes. Approximately 80% of all CS isolates encoded the iron transport genes, *sitABCD*, as well as the genes that encode the salmochelin and aerobactin siderophore systems (Figure 1C).

Using the genomic data, we grouped the *E. coli* into pathotypes based on their virulotypes (Figure 1D). We were only able to classify 43% of the EC isolates and 29% of BO isolates as ETEC and STEC, respectively. In contrast, over half of the CS isolates could be categorised as ExPEC, due to isolates sharing nine ExPEC VAGs (56%, *n* = 29/52).

Both EC and BO isolates had a small number of *E. coli* classified as STEC (3%) or ETECs (9%) within their groups, respectively. This was a particularly surprising outcome for the BO isolates as it is assumed that presence of the *stx2e* genes is the distinguishing factor for these *E. coli*. These groups also had a small number of *E. coli* harbouring both ETEC fimbriae and toxin genes, and the *stx2e* genes, indicating an ETEC/STEC mixed genotype (Figure 1D).

Finally, we were unable to pathotype just under a third of isolates (32%,) using the chosen VAGs (Figure 1D). We identified three (2.5%) EC isolates (189–250, 36–171, and 46–283) that encoded the intimin gene, *eae* and the gene for its receptor, *tir* (Pokharel et al., 2023). This was coupled with presence of genes for the type III secretion system suggesting these isolates were enteropathogenic *E. coli* (EPEC) (Supplementary Table 2). We also looked at the full complement of VAGs for the other non-typable isolates (*n* = 63, 30%), identifying a broad variety of genes associated with different pathotypes of *E. coli* which was outside the scope of this study (Supplementary Table 2).

### 3.3 Prevalence of AMR and MDR

A total of 61 different AMR genes were identified in 78.8% of isolates (Table 1). Isolates with AMR harboured between 1 and 19 AMR genes, which represented up to eight veterinary critically important and highly important antimicrobial (VCIA and VHIA) classes (World Organisation for Animal Health, 2021). There was no significant difference between any of the three groups encoding at least one AMR gene. AMR was identified in 83% of EC, 77% of CS and 68% of BO isolates. Full AMR genotype information can be found for each isolate included in this study in Table 1. AMR genotype information can be found in Supplementary Table 3.

**TABLE 1:** List of AMR genes detected in this study.

Gene	Antimicrobial class and Seqfinder variant ID	*N*				%		
		All *n* = 208	BO *n* = 34	EC *n* = 122	CS *n* = 52	BO	EC	CS
*aac(3)-IVa*	amino-g0020	28	4	23	1	11.8	18.9	1.9
*aadA15*	amino-g0094	2	0	2	0	0.0	1.6	0.0
*aadA2*	amino-g0098	54	6	43	5	17.6	35.2	9.6
*aadA24*	amino-g0103	2	1	1	0	2.9	0.8	0.0
*aadA25*	amino-g0104	1	0	1	0	0.0	0.8	0.0
*aadA5*	amino-g0108	4	1	3	0	2.9	2.5	0.0
*aadA*/*aadA10*	amino-g0110	2	0	2	0	0.0	1.6	0.0
*ant(3″)-1a*	amino-g0117	15	1	11	3	2.9	9.0	5.8
*ant(3″)-Ia*	amino-g0118	65	12	48	5	35.3	39.3	9.6
*aph(‘3)-Ia*	amino-g0136	16	5	11	0	14.7	9.0	0.0
*strA*	amino-g0139	67	10	33	24	29.4	27.0	46.2
*aph(3′)-Ic*	amino-g0140	3	0	3	0	0.0	2.5	0.0
*aph(3′)-IIa*	amino-g0142	5	2	3	0	5.9	2.5	0.0
*aph(4)-Ia*	amino-g0153	23	4	19	0	11.8	15.6	0.0
*strB*	amino-g0158	71	10	37	24	29.4	30.3	46.2
*bla*_CARB-2_	betaL-g0224	2	0	2	0	0.0	1.6	0.0
*bla*_CTX-M-32_	betaL-g0419	1	0	0	1	0.0	0.0	1.9
*bla*_DHA-1_	betaL-g0491	1	0	1	0	0.0	0.8	0.0
*bla*_HERA-3_	betaL-g0563	1	0	1	0	0.0	0.8	0.0
*bla*_TEM-1_	betaL-g1306	2	0	0	2	0.0	0.0	3.8
*ampP-ampC* promoter	betaL-g1999	2	0	2	0	0.0	1.6	0.0
*bla*_TEM-1b_	betaL-g2225	95	9	59	27	26.5	48.4	51.9
*bla*_TEM-1c_	betaL-g2226	4	1	3	0	2.9	2.5	0.0
*catA1*	chlor-g1554	6	1	4	1	2.9	3.3	1.9
*catA2*	chlor-g1562	8	0	1	7	0.0	0.8	13.5
*cml*	chlor-g1576	1	0	1	0	0.0	0.8	0.0
*cmlA1*	chlor-g1578	49	7	40	2	20.6	32.8	3.8
*floR*	chlor-g1585	13	2	6	5	5.9	4.9	9.6
*lnuF*	macro-g1722	10	2	6	2	5.9	4.9	3.8
*mefB*	macro-g1728	20	3	17	0	8.8	13.9	0.0
*mphA*	macro-g1731	5	1	4	0	2.9	3.3	0.0
*mphB*	macro-g1732	3	0	3	0	0.0	2.5	0.0
*mefC*	macro-g2353	1	0	1	0	0.0	0.8	0.0
*mphG*	macro-g2355	1	0	1	0	0.0	0.8	0.0
*lnuG*	macro-g2362	6	0	6	0	0.0	4.9	0.0
*gyrA* S83L mutation	quino-g1763	5	0	5	0	0.0	4.1	0.0
*oqxA1*	quino-g1767	1	1	0	0	2.9	0.0	0.0
*oqxB2*	quino-g1788	5	3	2	0	8.8	1.6	0.0
*qnrB19*	quino-g1831	1	0	1	0	0.0	0.8	0.0
*qnrB2*	quino-g1832	1	0	0	1	0.0	0.0	1.9
*qnrB4*	quino-g1852	1	0	1	0	0.0	0.8	0.0
*qnrS1*	quino-g1872	24	1	21	2	2.9	17.2	3.8
*sat2A*	strep-g1886	20	3	12	5	8.8	9.8	9.6
*sul2*	sulph-g1892	64	8	30	26	23.5	24.6	50.0
*sul3-1*	sulph-g1893	30	3	23	4	8.8	18.9	7.7
*sul3-2*	sulph-g2259	52	10	39	3	29.4	32.0	5.8
*tet-AB*	tetra-g1912	43	5	32	6	14.7	26.2	11.5
*tet-C*	tetra-g1915	2	0	2	0	0.0	1.6	0.0
*tet-M*	tetra-g1923	2	1	1	0	2.9	0.8	0.0
*tet-A-1*	tetra-g2310	7	1	4	2	2.9	3.3	3.8
*tet-A-2*	tetra-g2318	1	0	1	0	0.0	0.8	0.0
*tet-A-3*	tetra-g2320	80	12	44	24	35.3	36.1	46.2
*tet-A-4*	tetra-g2324	1	0	1	0	0.0	0.8	0.0
*dfrA1*	trime-g1937	29	4	18	7	11.8	14.8	13.5
*dfrA12*	trime-g1939	50	10	38	2	29.4	31.1	3.8
*dfrA14*	trime-g1941	19	0	6	13	0.0	4.9	25.0
*dfrA16*	trime-g1943	2	0	2	0	0.0	1.6	0.0
*dfrA17*	trime-g1944	5	1	4	0	2.9	3.3	0.0
*dfrA27*	trime-g1953	2	0	2	0	0.0	1.6	0.0
*dfrA5*	trime-g1961	6	1	1	4	2.9	0.8	7.7
*dfrA8*	trime-g1964	1	1	0	0	2.9	0.0	0.0
*dfrB1*	trime-g1966	1	0	0	1	0.0	0.0	1.9

Total (n) counts of each gene are shown in the table next to relative proportion (%). Percentages are shaded from white to red to distinguish from 1 to 100%. Antimicrobial classes: aminoglycosides (amino), *β*-lactamases (betaL), chloramphenicol (chlor), macrolide (macro), quinolones (quino), sulphonamides (sulph), tetracycline (tetra), and trimethoprim (trime). Seqfinder variant IDs have been included to demonstrate where different versions of the same AMR gene have been identified.

The most common antimicrobial classes across all isolates where a resistance genotype was identified were the aminoglycosides (64%), tetracyclines (60%) and sulphonamides (59%) (Figure 1E). A resistance genotype for trimethoprim was similar between *E. coli* associated with all three diseased states. Resistance genotypes were also similar between EC and CS associated *E. coli* for sulphonamides and tetracycline, whilst prevalence was approximately 10% lower in BO isolates to these antimicrobial classes. BO isolates also had much lower prevalence of resistance genotypes to the aminoglycosides and *β*-lactam antibiotics (Figure 1E).

Out of the 133 isolates that encoded resistance to one of the VCIA aminoglycosides, 13% isolates were positive for the *aac(3)-IVa* gene (gentamicin/apramycin/tobramycin resistance; Plattner et al., 2020; Figure 1E). The prevalence of this gene was higher in EC and BO groups (17%), compared with the CS group (~2%) (Table 1).

Eighteen percent of EC and BO groups had one of the five genes (*mefB*, *mefC*, *mphA*, *mphB* or *mphG*) encoding resistance to the VCIA macrolides azithromycin, erythromycin, spiramycin, and telithromycin (Table 1). These genes were absent in the CS group (Figure 1E). Ten percent of the enteric isolates carried genes associated with resistance for the VHIA, lincomycin (*inuF* and *inuG*). Three of these isolates also carried a VCIA macrolide gene as well, which was either *mphA* (isolate 20–187), or *mefB* (isolates 80–2 and 99–47).

Genes for fluoroquinolone resistance were predominantly identified in the EC group of *E. coli* (Figure 1E). The fluoroquinolone resistance gene *qnrS1* was predominantly identified in EC isolates (17%), with <4% of the BO and CS groups encoding this gene. A total of 4.1% of EC isolates also had mutations in the quinolone resistance determining regions (QRDRs) of the *gyrA* gene, giving rise to high level fluoroquinolone resistance. All five isolates (16–170, 20–187, 4–115, 57–120, 65–91) encoded the S83L mutation, with all but 57–120 also encoding a second substitution in D86N. These non-synonymous amino acid changes were not detected in any BO or CS isolates (Table 1).

We identified extended spectrum cephalosporinase (ESC) genes in <2% of isolates (Table 1). A CS associated *E. coli* harboured the *bla_CTX-M-32_* extended spectrum beta-lactamase (ESBL) gene, and an EC associated *E. coli* harboured the *bla_DHA-1_ ampC* resistance gene. Two additional ETEC isolates harboured mutation in the promoter of *ampC* gene associated with increased expression of β-lactamase.

The median number of antimicrobial classes where a resistance genotype was identified was four (Figure 1F). Comparing this metric across diagnosis, pathotype, and age exposed differences between some of the groups. This figure was highest in the ETEC (*n* = 6), and lowest in the STEC pathotype (*n* = 0). Both BO and neonatal groups had a slightly lower median of 3.

The proportion of all isolates that were MDR was 62% (Figure 1G). There was a statistically significant dependence between MDR and pathotype (*p* value = 7.29 × 10^−3^). The highest rate of MDR was seen in the ETEC pathotype (73%) and lowest in the STEC pathotype (29%). The odds of an ETEC isolate being MDR was 6.8 times higher than STEC (CI = 1.9, 25.1), and 2.7 times higher than ExPEC (CI = 0.4, 4.8).

### 3.4 Exploration of the phylogenetic relationships of *Escherichia coli* from different stages of pig production using SNP and K-mer based methods

We found that the average assembled genome size was approximately 5.1 Mbp (SD ± 0.29 Mbp), and from this, a core SNP alignment was generated using 102,329 SNPs present in the 208 *E. coli*. The core genome of these 208 *E. coli* consisted of approximately 4.1 Mbp (SD ± 0.1 Mbp), equating to 81% of the average full genome which contains approximately 4,000 genes (Blattner et al., 1997). There were an estimated 3,951 genes in the core genome, with 25,247 genes representing the pan-genome. A total of 19 STs were identified in ≥3 isolates (Supplementary Table 4), with the most common being ST10 (*n* = 27), ST1 (*n* = 15), ST772 (*n* = 13), ST90 (*n* = 13) and ST648 (*n* = 10).

We constructed a SNP based phylogenetic tree, which had on average, a core genome pairwise SNP distance (referred to as SNP distances from here on out) of 57,093 (SD ± 27,103) across the core genomes the 208 *E. coli* (Supplementary Table 4). However, SNP distances ranged from 8 to 102,329, highlighting that some isolates were likely clonal, whilst other were incredibly genetically diverse.

**Figure 2:**
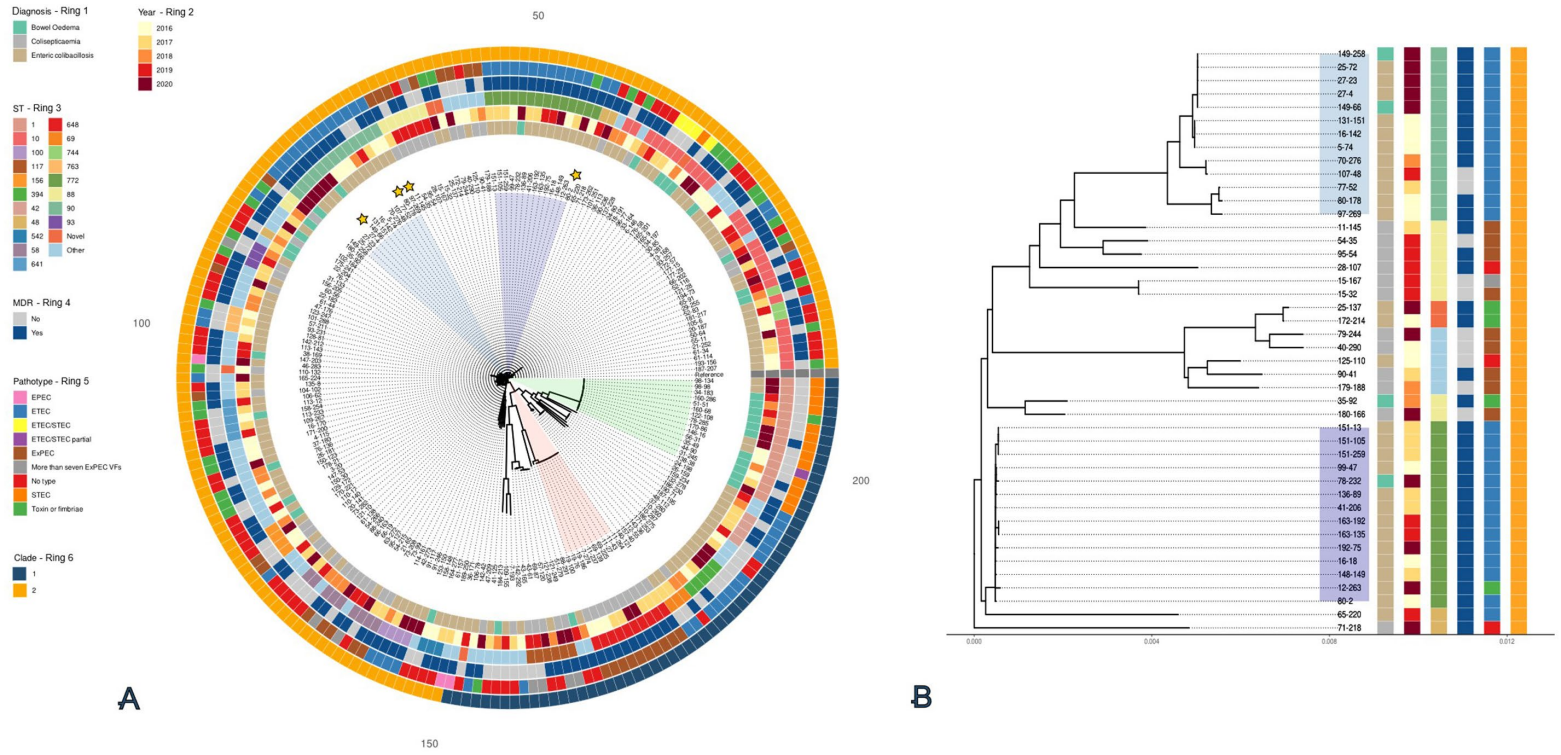
SNP based maximum likelihood tree with 208 *E. coli* isolated from diseased pigs **(A)**. The tree is based on the core alignment and inferred using RAxML-ng using the GTR + FO + G4m substitution model and rooted on the reference isolate (Reference: *E. coli* MG1655 U00096.3). Branches are drawn to scale in substitutions per site. A subtree **(B)** is shown for the 44 isolates that make up the subclades containing ST90 and ST772 as well as the surrounding isolates. Annotation for Diagnosis (Ring 1), Year (Ring 2), ST (Ring 3), MDR (Ring 4), Pathotype (Ring 5), and Clade (Ring 6) are all shown as coloured blocks around both trees. Shaded areas on the tree highlight key clusters for ST90 (blue), ST772 (purple), ST648 (red), and ST1 (green). Yellow stars highlight isolates that have undergone long-read sequencing.

We broadly split the tree into two large clades reflecting the branch lengths (Figure 2A). These clades were further subdivided by diagnosis (Figure 2A, ring 1), ST (Figure 2A, ring 3), MDR (Figure 2A, ring 4) and pathotype (Figure 2A, ring 5). We identified that 75% of the tree (*n* = 155/208) only had an evolutionary divergence of ~2.7% and were grouped into Clade 1, suggesting that much of the diversity in the tree was the result of a smaller number genetically diverse, isolates.

Subclades within Clade 2 included isolates from ST1 and ST648 (Figure 2A, red and green slices). ST1 (*n* = 15) contained all isolates that encoded the *stx2e* genes, with most in this group also associated with BO. This subclade had an average SNP distance of 414 SNPs (SD ± 268), showing that these were not clones but likely grouped together because they were more different than other isolates in the comparison. In contrast, ST648 which contained only MDR ExPEC (*n* = 10), had an average SNP distance of 89 SNPs (SD ± 13) suggesting that these isolates were more closely related, and likely share a more recent common ancestor (MRCA) than isolates in ST1.

Within Clade 1, we identified two of the largest clusters of predominantly EC associated, MDR, ETECs belonging to ST90 (*n* = 13) and ST772 (*n* = 14). From the tree we were able to identify that the mean percentage identity between their core genomes was 99.5%. The mean patristic distances within ST772 showed that this cluster was highly homogenous with an average SNP distance of 45 SNPs (SD ± 25, range = 8–143 SNPs). This compared to the average SNP distance of 844 SNPs (SD ± 855, range = 27–1,997 SNPs) within ST90, highlights that ST772 is far more homogeneous with some isolates likely being clonal. To support this, we were able to estimate from the tree that the MRCA between ST90 and ST772 lies at 0.0032 substitutions per site from the root, which corresponds to ~1.2% of the tree’s depth. Alongside this, the average SNP distances between ST90 and ST772 were 45,806 SNPs (SD ± 1,216, range = 41,371 – 47,282), demonstrating that although neighbouring each other, both form distinct subclades (Figure 2B).

To contextualise our dataset with *E. coli* from pigs during the production process, we generated a K-mer based distance matrix (Supplementary Table 5). We included data from this project with sequences from two other projects in the UK on *E. coli* from healthy pigs throughout the production process (*n* = 174; Storey et al., 2022), and pigs at slaughter (*n* = 248; AbuOun et al., 2020). This increased the total number of sequences compared in this study to *N* = 630.

Clustering based on STs, diagnosis, and phylogroup (Supplementary Table 5) were observed. A total of 48% (*n* = 305/630) fell into either phylogroup A or B2 associated with humans, whilst 26% (*n* = 162/630) fell into phylogroup B1, associated with domestic animals (Tenaillon et al., 2010). In total, there were 179 unique STs, with 26 STs occurring in ≥5 isolates. Interestingly, several of the predominant STs were only found in neonatal, pre- and post-weaned pigs from this study. This included the aforementioned ETEC associated ST90 (*n* = 13, phylogroup C; Supplementary Figure 1) and ST772 (phylogroup A; Supplementary Figure 2), as well as the ExPEC associated, ST648 (*n* = 10, phylogroup F, cluster 10).

### 3.5 Characterisation of the MDR plasmids carried by four isolates from ST90 and ST772

The high rates of MDR genotypes in isolates from ST90 and ST772 warranted further investigation. Long read sequencing of four MDR isolates from these STs was performed: 149–258 (BO, ST90), 80–178 (EC, ST90), 80–2 (EC, ST772), and 97–269 (EC, ST90; Supplementary Table 6). We identified between two and six plasmids encoding either virulence or AMR in these isolates. Plasmids carrying AMR genes had a greater size range (48–285 kb) than those carrying virulence genes (7–136 kb).

**Figure 3:**
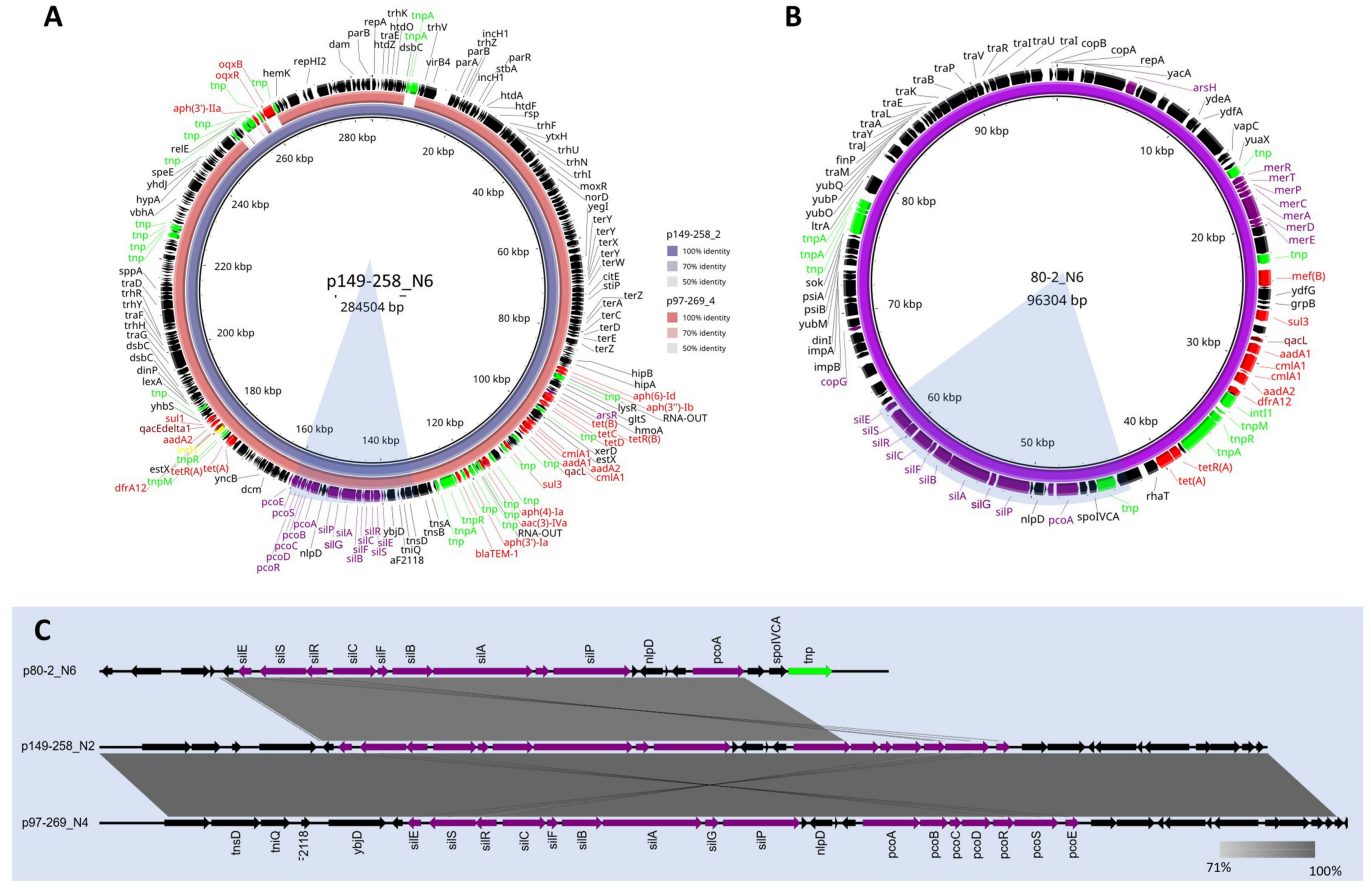
BLAST ring alignments of MDR plasmids containing the *sil* and *pco* operons from ST90 and ST772. Alignment **(A)** shows the comparison between the circular 284 kb plasmid reference, p149-258_N6 (inner ring), and the 273 kb plasmid, p97-269_N4 (outer ring). Identity between the two plasmids is indicated with opacity of the rings. The plasmid map of the circular, 96 kb plasmid, p80-2_N6, is shown in **(B)**. A linear alignment of the *sil* and *pco* MRG 30 kb regions of the p149-258_N6 and p97-269_N4, as well as the ~20 kb region in p80-2_N6 is shown in **(C)**. Shaded bars show percent identity (71—100%). The corresponding regions are highlighted in blue in **(A,B)**. Annotations for **A–C** shown and genes are coloured function: AMR (red), MRGs (purple), disinfectants (maroon), integrons (yellow), transposons (lime), and genes (black).

One MDR plasmid, p149-258_N2 (Figure 3A), was homologous to p97-269_N4, with both sharing 99% coverage and ~100% identity, although both parental isolates originated from different herds (149–258 and 97–269). Both plasmids encoded resistance to ampicillin (*bla*_TEM-1_), gentamicin (*aac(3)-IVa*), sulphonamides (*sul1* and *sul3*), tetracycline (*tet-AB*), and trimethoprim (*dfrA12*) (Figure 3A). Both only differed in a 10 kb region, present in 149–258_N2 but absent from 97–269_N4. It carried resistance genes for kanamycin (*aph(3′)-IIa*) and fluoroquinolones (*oqxB*). Both plasmids also carried the *silESRCFBAGP*, *pcoABCDRSE* and *qacL* genes which have been proposed to confer resistance to silver (Elkrewi et al., 2017), copper (Giachino and Waldron, 2020), and quaternary ammonium compounds (QAC) (Boyce, 2023), respectively. Some of these metal resistance genes (MRGs) were also identified in the MDR plasmid, p80-2_N6 (Figure 3B). These included *silESRCFBAGP* and *pcoA*. Aligning the MRG regions from these three plasmids (Figure 3C) showed that the gene synteny for the *sil* operon is the same across all three plasmids, but the *pco* operon in p80-2_N6 had been truncated. We also note the carriage of the ~4 kb *merRTPCADE* operon in p80-2_N6, which has been implicated in mercury detoxification (de Mattos D’Avila et al., 2024).

Both 80–2 and 149–258 carried a second plasmid harbouring AMR which can be viewed in Supplementary Figures 3A,B, respectively. The p80-2_N3 194kb plasmid from 80–2 was found to share 98% coverage and 97% identity with p80-178_N3 (Supplementary Figure 3A). Both p80-2_N3 and p80-178_N3 carried resistance for the VCIAs, apramycin and gentamicin (*aac(3)-IVa*) in a class I integron. The second AMR plasmid from 149-258 (Figure 3B), was a smaller 77 kb plasmid (p149-258_N6), that encoded three AMR genes for aminoglycoside resistance, *aph(4)-la*, *aac(3)-IVA*, and *aph(3′)-la*. It also encoded resistance for *β*-lactams (*bla*_TEM-1_). All AMR and virulence plasmids are listed in Supplementary Table 6 along with their replicon types.

## 4 Discussion

Juvenile pigs, including neonatal, pre- or post-weaning pigs, are particularly susceptible to EC, BO or CS diseases, due to factors such as under-developed immune systems, hygiene and the environment. As the risk of disease is a major concern, control measures for pathogenic *E. coli* in pigs are wide-ranging and include various practices such as: ensuring excellent hygiene; pig management and environment; producing robust healthy pigs at weaning; manipulation of diets; ensuring adequate colostrum intake; weaning at a later age; and use of probiotics (Pan et al., 2017). The use of live oral vaccines for different VAGs such as F4 and the Stx2e toxin, has also been used as a control measure (Johansen et al., 1997; Melkebeek et al., 2013).

The genomes of the 208 *E. coli* analysed in this study reflect what has been reported previously where the core genome of *E. coli* is somewhere around 4,000 genes (Geurtsen et al., 2022) and the pan-genome is estimated to exceed 22, 000 (Robins-Browne et al., 2016). Isolates predominantly clustered broadly into phylogroups A, B1, and C, with the latter two commonly associated with livestock in the EU (Kaspersen et al., 2024). Our SNP based analysis showed that there was also a significant amount of genetic variability within these isolates, with some STs having more genetic relatedness.

Despite high core-genome variability, these isolates could be pathotyped using relatively few genetic features that have been reported in the literature. The more homogenous ExPECs (~70% of total) carried VAGs for biofilm production, adherence, iron acquisition, serum survival, the haemolysin toxin and the colicinV bacteriocin. These results are also comparable to reports from other geographic locations (Tan et al., 2011), and likely reflect adaptations facilitating the colonisation by ExPECs of multiple anatomical sites (Russo and Johnson, 2009).

The InPEC were more challenging to categorise using the current pathotyping schemes for ETEC and STEC (Luppi, 2017), due to the absence of well-characterised virulence genes in several isolates in our panel. There were especially few VAGs in the BO group, where only 35% of *E. coli* encoded both genes required for a functional Stx2e toxin, which is characteristically required for the development of bowel oedema in pigs. For the remaining 65% of *E. coli* where *stx2e* was absent but the pigs were diagnosed with BO, it’s possible a mixed infection occurred with both STEC and ETEC, resulting in only the ETEC being cultured. Although there is possibility that other unknown VAGs were present in these cases.

In fact, although the F18 fimbriae has been found in ETEC associated with PWD (Garcia et al., 2020), we identified a statistically significant association between presence of genes encoding the F18 and the Stx2e toxin. This supports previous reports where the Stx2e toxin was almost exclusively found associated with the F18 fimbriae (Luppi et al., 2016).

However, the most common VAGs harboured by ETECs in our study were the F4 (33%) fimbriae and STb (48%) toxin. This mirrors what has been observed in Europe from pigs with diarrhoeal diseases (Luppi et al., 2016). For the isolates that we could not pathotype (32%), uncommon VAGs such as those encoding EaeH and Paa were detected in some isolates and possibly play a role in their pathogenicity. As the virulence mechanisms behind these VAGs are still poorly understood (Ngeleka et al., 2003; Sheikh et al., 2014), further work is required in this area.

We were able to validate our primary screening method using this WGS dataset. One of the main limitations of qPCR is the need for previously characterised genes (Anjum, 2015). We demonstrate that our qPCR method is ~97% accurate, highlighting its accuracy when compared to WGS. However, we do not gain any additional information outside of the nine virulence genes included. Characterising the isolates in this study has highlighted some possible ‘blind-spots’, namely genes such as *stb*, which was commonly found in ETECs. Additionally, we show that the majority of ETEC and STEC in this study carried genes encoding either F4 or F18, so some of the genes used in this screen may be considered redundant if they are uncommon. Future work will look at using this genomic data to improve our current methods and broaden the scope of our scanning surveillance on pathogenic *E. coli* to ensure our front-line methods are beneficial to veterinarians requesting these tests.

AMR was present in approximately 80% of *E. coli* from this study. The InPEC predominantly harboured genes encoding resistance for gentamicin (17%) and azithromycin (10%), compared to CS associated *E. coli*, where only ~2 and 0% of isolates, respectively, carried the resistance genes. EC associated *E. coli* predominantly encoded (17%) the fluoroquinolone resistance gene *qnrS1*, compared to BO and CS isolates (<4%). EC associated *E. coli* were also the only isolates (4.1%) to harbour mutations in the QRDR that provide resistance to the fluoroquinolones.

MDR genotypes were identified in 62% of the 208 *E. coli* in this study. Due to differences in the sampling strategy with previous studies, it is hard to make direct comparisons, however, the MDR rate in these *E. coli* was higher than those detected in healthy adult pigs at slaughter (46.9%) (AbuOun et al., 2020), and significantly higher than those from healthy pigs throughout the production cycle from a farm with history of low antimicrobial usage (3.9%) (Storey et al., 2022). These results suggest that pathogenic *E. coli* isolated from sick juvenile pigs could be a significant reservoir of AMR.

Nevertheless, the predominant MDR genotypes in our *E. coli* were to commonly used antimicrobials such as tetracycline, the penicillin *β*-lactams, sulphonamides, and trimethoprim (Fairbrother and Nadeau, 2019). Genotypic resistance to the HP-VCIAs and HP-CIAs, such as aminoglycosides, macrolides, and the fluoroquinolones were <21%; ESC genes were only detected in ~2% of isolates. These figures likely reflect the very low use of HP-CIAs (0.01%) in pigs in the UK (Broadfoot et al., 2023).

Further investigation demonstrated that MDR, particularly within the ETEC group, was 6.8 times and 2.7 times more likely than in STEC and ExPEC groups, respectively. To investigate this, we carried out a phylogenetic analysis of all 208 *E. coli*. Within Clade 1, we were able to identify two larger, genetically distinct clusters belonging to ST90 and ST772, that were predominantly characterised as ETECs with MDR genotypes. The over-representation of these STs when compared to other STs provides an explanation for the increased likelihood of MDR in the ETEC. Both STs have previously been reported in Danish and Belgian pig herds (Garcia et al., 2020; De Koster et al., 2023; De Koster et al., 2021), but we were unable to find any reports of these STs within herds in England. With limited survey data on pathogenic *E. coli* from this age bracket of pigs in England, it is possible that these lineages have been underreported. In support of this, a K-mer based comparison of the genomic data in this study with two other datasets from the APHA on healthy pigs at five different ages (Storey et al., 2022), and adult pigs at slaughter (AbuOun et al., 2020), we were able to show that ST90 and ST772 were only assigned to InPEC from diseased juvenile pigs.

Further investigations into the AMR MGEs carried by some isolates from ST90 and ST772 showed homologous plasmids in isolates from both STs. Additionally, three plasmids carried the *sil* and *pco* operons, reported to provide resistance against silver and copper (Munson et al., 2000), respectively. The carriage of metal resistance and AMR genes has been reported in bacteria, especially those associated with agriculture (Pal et al., 2015). However, it is unclear why proposed silver resistance genes would be co-localised on plasmids alongside MDR, as silver is not associated with agricultural pollution (Purcell and Peters, 1998). Previous reports have shown that the *sil* operon is cryptic in most isolates of *E. coli*, and it is switched on through spontaneous missense mutations in the *silS*, sensor kinase gene (Elkrewi et al., 2017). This raises the question that the *sil* operon could be functionally involved in an unknown process, but whether this may provide a selective advantage to *E. coli* in this niche is currently unclear.

To conclude, in this retrospective study of pathogenic *E. coli* isolated from diseased pigs in England, we show common features in *E. coli* originating from pigs with one of the three recognised *E. coli* associated disease presentations. We were able to use this data to validate our current front-line methods and highlight our surveillance ‘blind-spots’ for pathogenic *E. coli* affecting juvenile pigs. Additionally, the genetic diversity in the core genomes of these isolates was found to be quite high, with characteristics for virulence and MDR identified in high-priority lineages such as ST90, ST648, ST772. We found these STs were uniquely isolated from diseased juvenile pigs. Analysis of some of their MGEs identified several MDR plasmids, co-linked with heavy metal resistance genes as well as resistance genes for disinfectants such as QACs. The role these additional factors may play in the spread of AMR is still unclear, but there should be further investigation into these high-priority lineages due to their potential for causing disease and acting as reservoirs for AMR within the ecological niche of the pig industry. With this information, we will be able to develop more robust surveillance methods, provide better support for front-line veterinary workers to improve animal welfare, and reduce the impact of these diseases on swine production.

## Data Availability

The datasets presented in this study can be found in online repositories. The names of the repository/repositories and accession number(s) can be found in the article/Supplementary material.

## Footnotes

^1^ https://www.bioinformatics.babraham.ac.uk/projects/fastqc/

^2^ https://github.com/tseemann/abricate

^3^ https://www.gov.uk/guidance/animal-disease-scanning-surveillance-at-apha
