# Supplementary material for: Diversity of antimicrobial resistance and virulence genes of pathogenic Escherichia coli recovered from pigs in England
Source: Front Microbiol. 2025 Oct 22;16:1668327. doi: 10.3389/fmicb.2025.1668327 (PMC12586179; doi:10.3389/fmicb.2025.1668327)
Supplement: Supplementary file 2 [file Data_Sheet_1.docx]

Supplementary Materials

ETEC characteristically express fimbriae that allow them to adhere to enterocytes in the epithelial layer of the gut. ETEC strains then need to express one or more enterotoxins that can induce diarrhoea. In this SOP, we describe a rapid real-time PCR method that does not require a gel-based step to identify eight different genes associated with ETEC, using TaqMan probes. The assay target either:

- fanA, fim41a, faeG (Multiplex 1)
- stx2e, eltA and sta1, (Multiplex 2)
- and fedA, fasA, and f17A (Multiplex 3).

Each multiplex also includes and internal gadA control for specifically identifying E. coli.

In the tables below, are details of the:

- Controls
- Multiplexes
- PCR conditions
- Oligos, probes and their concentrations
- Reaction set up.

Supplementary materials Table 1. Control strains used in this real-time PCR and their origin

| **Strain** | **Origin** |
| --- | --- |
| Y05209 | APHA UK porcine |
| B1305 | APHA Bacti collection used for microarray work originally |
| Abbotstown | In Bacti collection used for microarray work originally Available of NCTC 10964 |
| HM1535 | VLA B1299 in Bacti collection used for microarray work originally |
| Y05176 | APHA UK porcine |
| Y05053 | APHA UK porcine |

Supplementary materials Table 2. List of multiplexes, genes, targets and their related characteristics for each of the control strains.

| **Multiplex** | **Gene** | **Target** | **Characteristic** | **Positive** *E. coli* **control** |
| --- | --- | --- | --- | --- |
| Multiplex 1 | *faeG* | F4 | Fimbriae | Abbotstown |
|  | *fanA* | F5 |  | Y05209 |
|  | *fim41a* | F41 |  | Y05209/B1305 |
| Multiplex 2 | *sta1* | STa | Toxin | Y05209/HM1535/Y05053 |
|  | *eltA* | LTa |  | Abbotstown |
|  | *stx2e* | Stx2e |  | V97 |
| Multiplex 3 | *f17A* | F17a | Fimbriae | Y05176 |
|  | *fedA* | F18 |  | Y05053 |
|  | *fasA* | F6 |  | HM1535 |

Supplementary materials Table 3. Conditions for the real-time PCR reaction.

| Stage | Temperature (oC) | Duration (mm:ss) | Cycles |  |
| --- | --- | --- | --- | --- |
| Hot Start | 95 | 15:00 | 1 |  |
| Denature | 94 | 00:20 | 36 |  |
| Anneal | 55 | 00:20 |  |  |
| Extension | 72 | 00:30 |  | 🡨 Enable data collection |

Supplementary materials Table 4. Oligo sequences used in the real-time PCR.

| **Oligos** | **Sequence** |
| --- | --- |
| F4 Fwd | GGTTCAGTGAAAGTCAATGCATCT |
| F4 Rev | CCCCGTCCGCAGAAGTAAC |
| F5 Fwd | GCTATTAGTGGTCATGGCACTGTAG |
| F5 Rev | TTTGTTTTCGCTAGGCAGTCATTA |
| F6 Fwd | CCAAAGTATTCCACTGCAAGCA |
| F6 Rev | GCCGTAACTCCACCGTTTGT |
| F17 Fwd | GCAGAAAATTCAATTTATCCTTGGAA |
| F17 Rev | TAATTGTACCGTCATAAGCAAGC |
| F18 Fwd | TTGTGCTTCCTTGTCCAATAAAAC |
| F18 Rev | CTCCCCCTTGATTAGCAAAACC |
| F41 Fwd | CTGCTGATTGGACGGAAGGT |
| F41 Rev | CCAGTCTTCCATAGCCATTTAACAG |
| LTa Fwd | CCGGCAGAGGATGGTTACAG |
| LTa Rev | GAATCCAGGGTTCTTCTCTCCAA |
| STa Fwd | GCAAAATCCGTTTAACTAATCTCAAA |
| STa Rev | ACAGAAATAAAAATTGCCAACATTAGC |
| Stx2e Fwd | CAGTACAACGCGCCACACTT |
| Stx2e Rev | AATCAGTACCAGACCCGGCG |
| GadA Fwd | ACCGACATCGTGGTGATGC |
| GadA Rev | AGCAACAGTTCAGCAAAGTCCA |

Supplementary materials Table 5. Probe sequences and their respective fluorophore and quencher.

| **Oligo Name** | [**5' Reporter / 3' Quencher Combination**](file:///C:\Users\x953767\AppData\Local\Microsoft\Windows\INetCache\Content.MSO\2B1793C4.xlsx#'Ordering Codes'!A1) | **Sequence** |
| --- | --- | --- |
| F4 Probe | Cyanine5 / BHQ-2 | CCACCTCTCCCTAACACACCGGCAT |
| F5 Probe | 6-FAM / TAMRA | ATTTTAAACTAAAACCAGCGCCCGGCA |
| F6 Probe | HEX / TAMRA | ACATCGGAACCACCACAGGGAATCCT |
| F17 Probe | Cyanine5 / BHQ-2 | GGCGGCTGCGTCATCTTCTGCT |
| F18 Probe | 6-FAM / TAMRA | TTTCGGTTAACTGCCCGCTCCAAGT |
| F41 Probe | HEX / TAMRA | TGATGTAATTTCACCACCAATAATAATGTCACCTG |
| LTa Probe | HEX / TAMRA | TAGCAGGTTTCCCACCGGATCACC |
| STa Probe | Cyanine5 / BHQ-2 | TTACCTCCCGTCATGTTGTTTCACGGAT |
| Stx2e Probe | 6-FAM / TAMRA | TCCCTGGCTCGCTTCTGCGGGCCT |
| GadA Probe | ROX / BHQ-2 | CATTATGTGTCGTCGCGGCTTCGAA |

Supplementary materials Table 6. Reaction set up for the real-time PCR for a single reaction for each of the multiplexes.

| Oligo/ probe | Working stock Concentration (µM) | MP1 | MP2 | MP3 | x1 (µL) |
| --- | --- | --- | --- | --- | --- |
| Fwd | 20 | F4 (K88) | STa | F6 (987p) | 0.5 |
| Rev | 20 | F4 (K88) | STa | F6 (987p) | 0.5 |
| Prb | 5 | F4 (K88) | STa | F6 (987p) | 0.5 |
| Fwd | 20 | F5 (K99) | LTa | F17 | 0.5 |
| Rev | 20 | F5 (K99) | LTa | F17 | 0.5 |
| Prb | 5 | F5 (K99) | LTa | F17 | 0.5 |
| Fwd | 20 | F41 | Stx2e | F18 | 0.5 |
| Rev | 20 | F41 | Stx2e | F18 | 0.5 |
| Prb | 5 | F41 | Stx2e | F18 | 0.5 |
| Fwd | 2 | GadA | GadA | GadA | 0.5 |
| Rev | 2 | GadA | GadA | GadA | 0.5 |
| Prb | 5 | GadA | GadA | GadA | 0.5 |
|  |  | Master mix | Master mix | Master mix | 12.5 |
|  |  | Water | Water | Water | 4 |
|  |  | DNA | DNA | DNA | 2.5 |
| Total |  |  |  |  | 25 |

Supplementary Table 7. Threshold criteria for determining a positive result using the real-time PCR.

| Criteria for determining a positive result using the ETEC virulence RT-PCR | |
| --- | --- |
| Criteria 1 | All controls have the expected results with Cq values between 10-25 and cross a background threshold of 200 (ΔR). |
| Criteria 2 | A sample shows a positive result in all three multiplexes for the internal, gadA control gene. |
| Criteria 3 | A positive Cq value occurs between 10-30 and crosses a background threshold of 200 (ΔR). |
| Criteria 4 | The Cq values between a positive result for a gene and that of the internal control do not differ by ±5 Cq values. |
