# Supplementary figures and images for: Diversity of antimicrobial resistance and virulence genes of pathogenic Escherichia coli recovered from pigs in England
Source: Front Microbiol. 2025 Oct 22;16:1668327. doi: 10.3389/fmicb.2025.1668327 (PMC12586179; doi:10.3389/fmicb.2025.1668327)

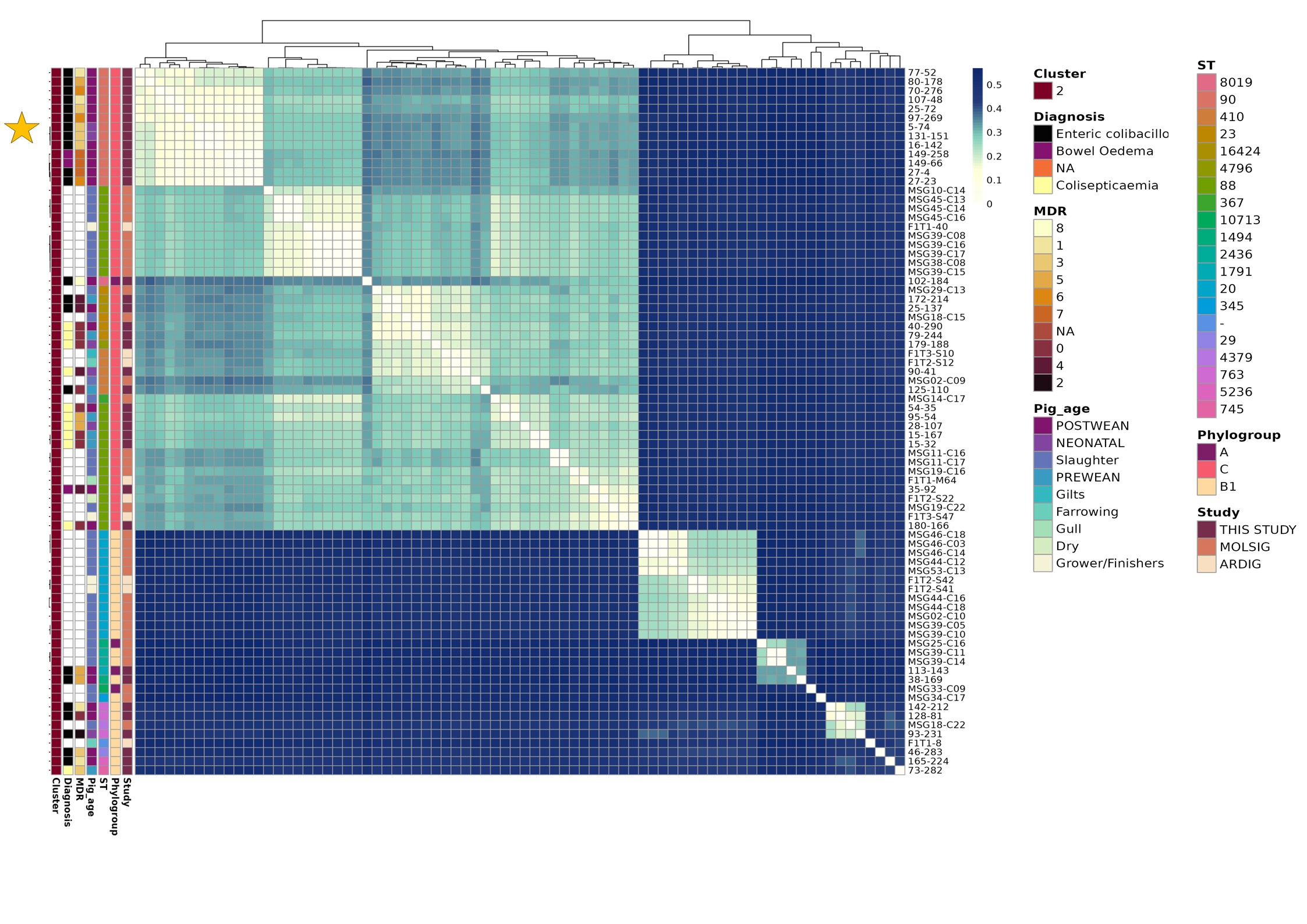

Supplement: Supplementary file 3 [file Image_1.jpeg]

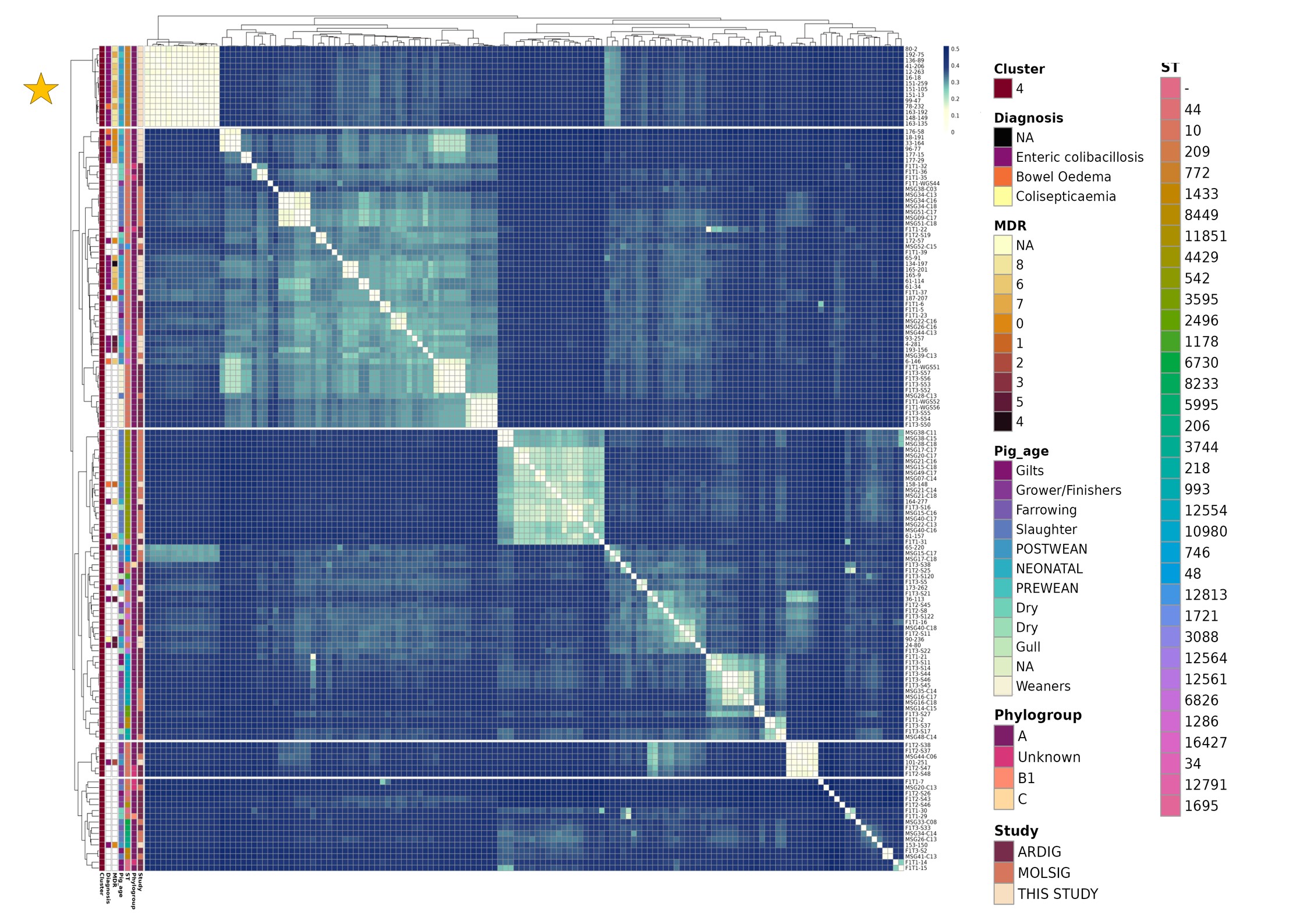

Supplement: Supplementary file 4 [file Image_2.jpeg]

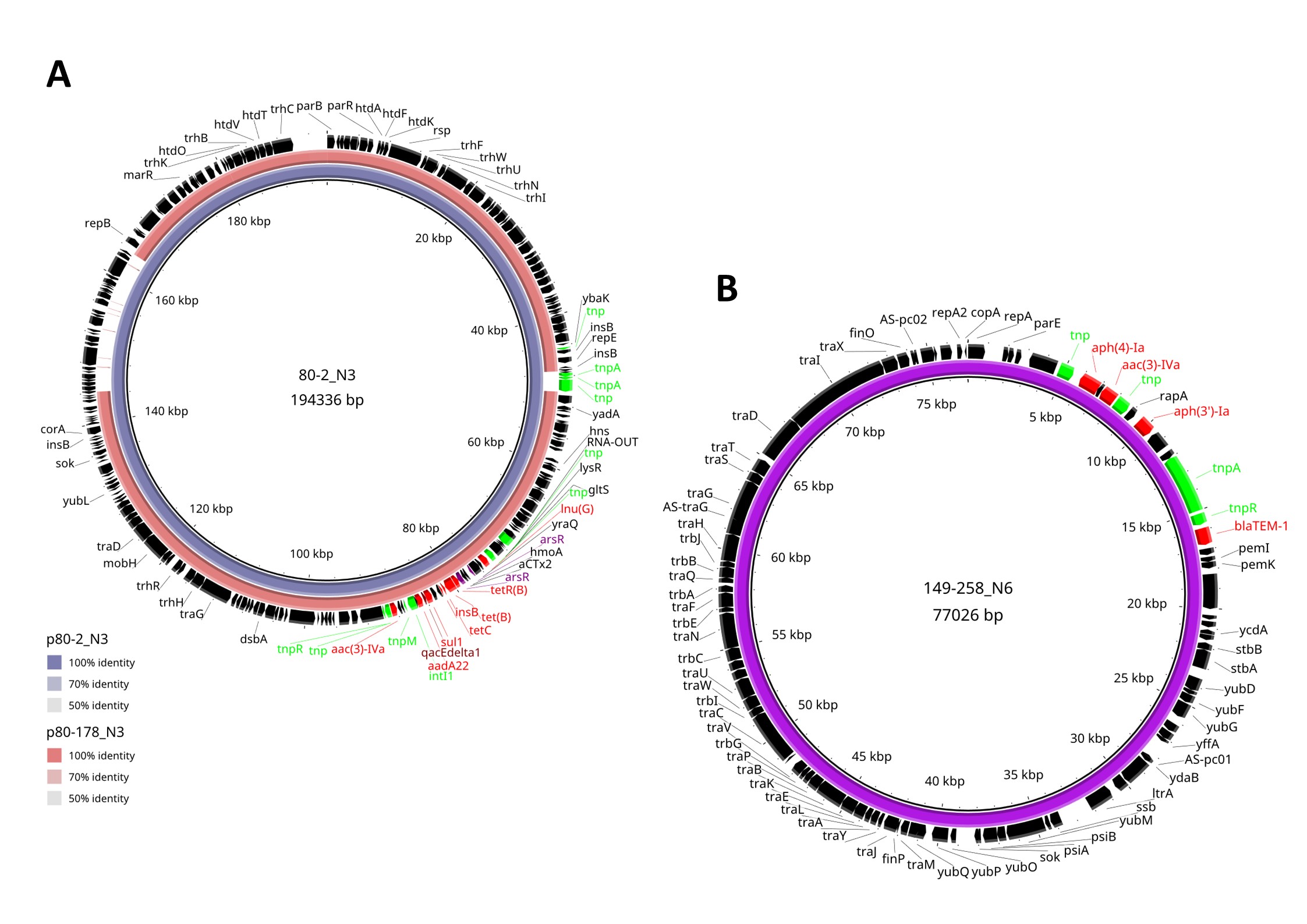

Supplement: Supplementary file 5 [file Image_3.jpeg]
